# Structural heterogeneity assessment among the isoforms of fungal 1-aminocyclopropane-1-carboxylic acid (ACC) deaminase: a comparative in silico perspective

**DOI:** 10.1186/s43141-021-00294-0

**Published:** 2022-02-01

**Authors:** Krishnendu Pramanik, Narayan Chandra Mandal

**Affiliations:** Mycology and Plant Pathology Laboratory, Department of Botany, Siksha Bhavana, Visva-Bharati, Santiniketan, Birbhum, West Bengal PIN – 731 235 India

**Keywords:** ACC deaminase, Fungi, Phylogeny, Protein functional annotation, Structural analyses

## Abstract

**Background:**

The primary amino acid sequence of a protein is a translated version from its gene sequence which carries important messages and information concealed therein. The present study unveils the structure-function and evolutionary aspects of 1-aminocyclopropane-1-carboxylic acid deaminase (ACCD) proteins of fungal origin. ACCD, an important plant growth-promoting microbial enzyme, is less frequent in fungi compared to bacteria. Hence, an inclusive understanding of fungal ACC deaminases (fACCD) has brought forth here.

**Results:**

In silico investigation of 40 fACCD proteins recovered from NCBI database reveals that fACCD are prevalent in *Colletotrichum* (25%), *Fusarium* (15%), and *Trichoderma* (10%). The fACCD were found 16.18–82.47 kDa proteins having 149–750 amino acid residues. The enzyme activity would be optimum in a wide range of pH having isoelectric points 4.76–10.06. Higher aliphatic indices (81.49–100.13) and instability indices > 40 indicated the thermostability nature. The secondary structural analysis further validates the stability owing to higher α-helices. Built tertiary protein models designated as ACCNK1–ACCNK40 have been deposited in the PMDB with accessions PM0083418–39 and PM0083476–93. All proteins were found as homo-dimer except ACCNK13, a homo-tetramer.

**Conclusions:**

Hence, these anticipated features would facilitate to explore and identify novel variants of fungal ACCD in vitro aiming to industrial-scale applications.

**Supplementary Information:**

The online version contains supplementary material available at 10.1186/s43141-021-00294-0.

## Highlights

• First comprehensive in silico annotation of fungal ACC deaminases (fACCD).

• *Colletotrichum*, *Fusarium*, and *Trichoderma* are predominant to possess fACCD.

• fACCD are 16.18–82.47 kDa proteins with optimal pH between 4.76 and 10.06.

• Majority are thermostable with higher aliphatic indices and instability indices < 40.

• fACCD are found as homo-dimer except ACCNK13, a homo-tetramer.

**Graphical Abstract:**

**Supplementary Information:**

The online version contains supplementary material available at 10.1186/s43141-021-00294-0.

## Graphical abstract

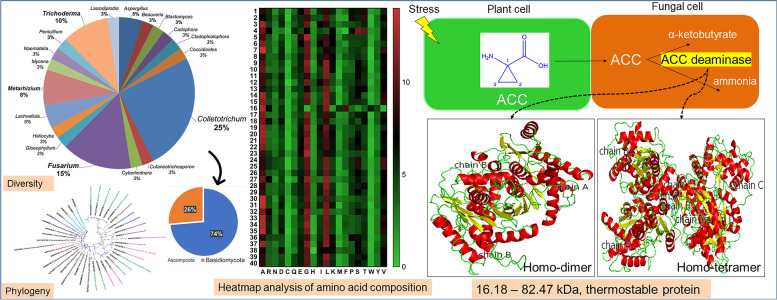

## Background

Ethylene, a volatile phytohormone synthesized from methionine through two intermediates viz. S-adenosyl-L-methionine (SAM) and 1-aminocyclopropane-1-carboxylic acid (ACC) [1]. It is known to be involved in regular plant growth and development including seed germination, fruit ripening, flowering, and senescence [2]. The phytohormone, on the other hand, is known to overproduce (known as “stress ethylene”) as a response to biotic and abiotic stresses that lead to altered plant growth and development, often leading to death [3]. This happens as a rapid surge of ACC (the immediate precursor of ethylene) levels in plant cells either during the interaction with phytopathogens [3] or exposure to abiotic stresses like heavy metal, drought, salinity, etc. [4, 5]. The influence of pathogen-induced ethylene in virulence and disease development has been studied earlier [6, 7].

A group of microorganisms possesses 1-aminocyclopropane-1-carboxylic acid deaminase (ACCD) [EC 3.5.99.7] activity that plays a major role in ethylene signaling in plants. The microbial ACCD cleaves ACC of plant cells into α-ketobutyrate and ammonia during the synergistic plant-microbe interaction under stress and drops the “stress ethylene” levels in plants thereby assisting in normal plant functioning. The ACCD is known to be prevalent in bacteria, and in some beneficial fungi as well as in stramenopiles [8]. However, the study of ACCD of fungal origin is less compared to others [9]. Interestingly, the ACCD is also evident in some plant pathogenic species of *Alternaria*, *Aspergillus*, *Colletotrichum*, and *Fusarium* that suggests the likely role of ACCD in the ecological fitness of the fungi [3]. Ethylene perception was necessary during the spore germination and appressorium formation in *Colletotrichum gloeosporioides* while in *Botrytis cinerea*, the hormone exaggerated the transcriptional reprogramming of the genes associated with plant interaction [10]. Like plant growth-promoting rhizobacteria (PGPR), ACC deaminase is also evident in plant growth-promoting fungi (PGPF) such as in several *Trichoderma* species [9, 11]. Still, the distribution of ACCD in fungal species is limited which consequently limits our understanding of the structure-function aspects of the fungal ACCD (fACCD). Hence, the overall structural as well as functional features of fACCD are to be explored to facilitate the process of discovery of more novel variants ACCD from different fungal classes.

The lack of fundamental structural features including three-dimensional structure of a protein of interest discernibly limits the knowledge of biological function. While an x-ray crystallography or at least a nuclear magnetic resonance (NMR) produces an accurate structural feature of a particular protein, it is often not accessible and feasible as well especially for screening large set of proteins. Besides, few proteins also lose to sustain their native state due to chemical properties and technical limitations that suggests predictive approaches to adopt straightaway as a complement of wet-lab set up [12]. The present study was undertaken to unravel the structural, functional, and phylogenetic perspectives of known fungal ACC deaminase that is often encoded by the gene acdS. To date, to the best of our knowledge, there is no in-depth investigation on fungal ACC deaminase that needs to be examined. Here, some open-source bioinformatic tools, web-servers, and offline tools were utilized to analyze the linear chain of amino acids that is the principal source of information hidden therein. Starting from the phylogenetic analysis, a thorough physicochemical characterization, secondary structural conformations were derived followed by representations of tertiary structural arrangements. This is accompanied by structural validation to assess the quality of structures and functional analysis was also targeted to find the conserved residues in the proteins of interest. In the end, we have submitted the built 3-D models of fACCD proteins in public repositories for further research.

## Methods

### Amino acid sequence recovery

The amino acid sequences of different fungal ACCD proteins (fACCD) were extracted from The National Center for Biotechnology Information (https://www.ncbi.nlm.nih.gov/). The proteins mentioned therein as “hypothetical proteins,” “probable ACC deaminase,” and “unnamed protein product” were screened out to keep away from any ambiguity in selecting appropriate protein sequences. These sequences were saved in FASTA format for examination through bioinformatic analyses.

### Phylogeny of fungal ACCD

Evolutionary relationship based on the fACCD proteins among the selected taxa was inferred in MEGA X [13] using the Neighbor-Joining method [14] with 1000 bootstraps. The evolutionary distances were computed using the Poisson correction method [15] and are in the units of the number of amino acid substitutions per site. All ambiguous positions were removed for each sequence pair using the pairwise deletion option.

### Physicochemical characterization

The primary sequence analyses for all selected fACCD proteins were executed by determining the computation of various physical and chemical parameters from ExPASy ProtParam tool [16]. This tool (https://web.expasy.org/protparam/) analyses length of sequence, amino acid composition, molecular weight (MW), isoelectric point (pI), extinction coefficient (EC), instability index (II), aliphatic index (AI), grand average of hydropathicity (GRAVY), and the total number of negatively as well as positively charged residues (TNR and TPR respectively).

### Secondary structure prediction

Prediction of protein folding was performed in the improved self-optimized prediction (SOPMA) method [17] to determine the percentage of α-helices, extended strands, β-turns, and random coils for the fACCD proteins.

#### Template selection and homology-based modeling

All selected fACCD proteins were used to determine the 3-D model for each protein structure. SWISS-MODEL, a homology-based protein modeling server [18] was used to predict the protein structures in the following order: template search>template selection>model building. The SWISS-MODEL template library (SMTL version 2020-11-04, PDB release 2020-10-30) was searched with BLAST [19] and HHBlits [20] for evolutionary related structures matching the target sequences. Suitable templates for each target protein were chosen tactically from the 50 templates obtained per search based on sequence similarity, query coverage, global model quality estimation (GMQE), and quaternary structure quality estimation (QSQE). Hence, one particular template per protein was selected based on target-template alignment to build final protein models using ProMod3 3.1.1. The predicted structures were visualized in the open-source PyMOL 1.3 software.

### Structure assessment

Evaluation of 3-D structures was performed following SWISS Model structure assessment project (https://swissmodel.expasy.org/assess) followed by the structure analysis and verification server (SAVES v6.0) which determines the stereochemical quality of a protein structure by evaluating residue-by-residue geometry as well as overall structural geometry [21].

### Model deposition

The built 3-D protein models were deposited to the protein model database (PMDB) which is a public resource for storing protein models to give access as well as validating experimental data [22]. PMDB database (http://srv00.recas.ba.infn.it/PMDB/) assigns a unique identifier for each submitted model to directly access the relevant data.

### Functional analyses

To find out conserved domains among the fACCD proteins, a multiple sequence alignment program, Clustal Omega (https://www.ebi.ac.uk/Tools/msa/clustalo/), was used which generate alignments for more than three sequences [23]. Additionally, a motif finder tool (https://www.genome.jp/tools/motif/) was used to find common motifs among the selected proteins.

## Results

### Amino acid sequence recovery

A sum of 40 ACCD protein sequences of fungal origin (fACCD) which had a clear description in NCBI as compared to others was selected for comprehensive in silico investigation to represent an overview on fungal ACC deaminase. Those 40 fACCD protein sequences belonged to 19 fungal genera which are dominated by *Colletotrichum* (25%), *Fusarium* (15%), and *Trichoderma* (10%) followed by *Metarhizium* (8%) (Fig. S1). Other fungal genera include *Lachnellula* (5%), *Aspergillus* (5%), *Beauveria* (3%), *Blastomyces* (3%), *Cadophora* (3%), *Cladophialophora* (3%), *Coccidioides* (3%), *Cutaneotrichosporon* (3%), *Cyberlindnera* (3%), *Gleophyllum* (3%), *Heliocybe* (3%), *Mycena* (3%), *Naematelia* (3%), *Lasiodiplodia* (3%), and *Penicillium* (3%) (Fig. S1). Remarkably, the fungal taxa mentioned herein were restricted to the Ascomycota (74%) and Basidiomycota (26%) (Fig. S2).

### Phylogeny of fungal ACCD

**Fig. 1:**
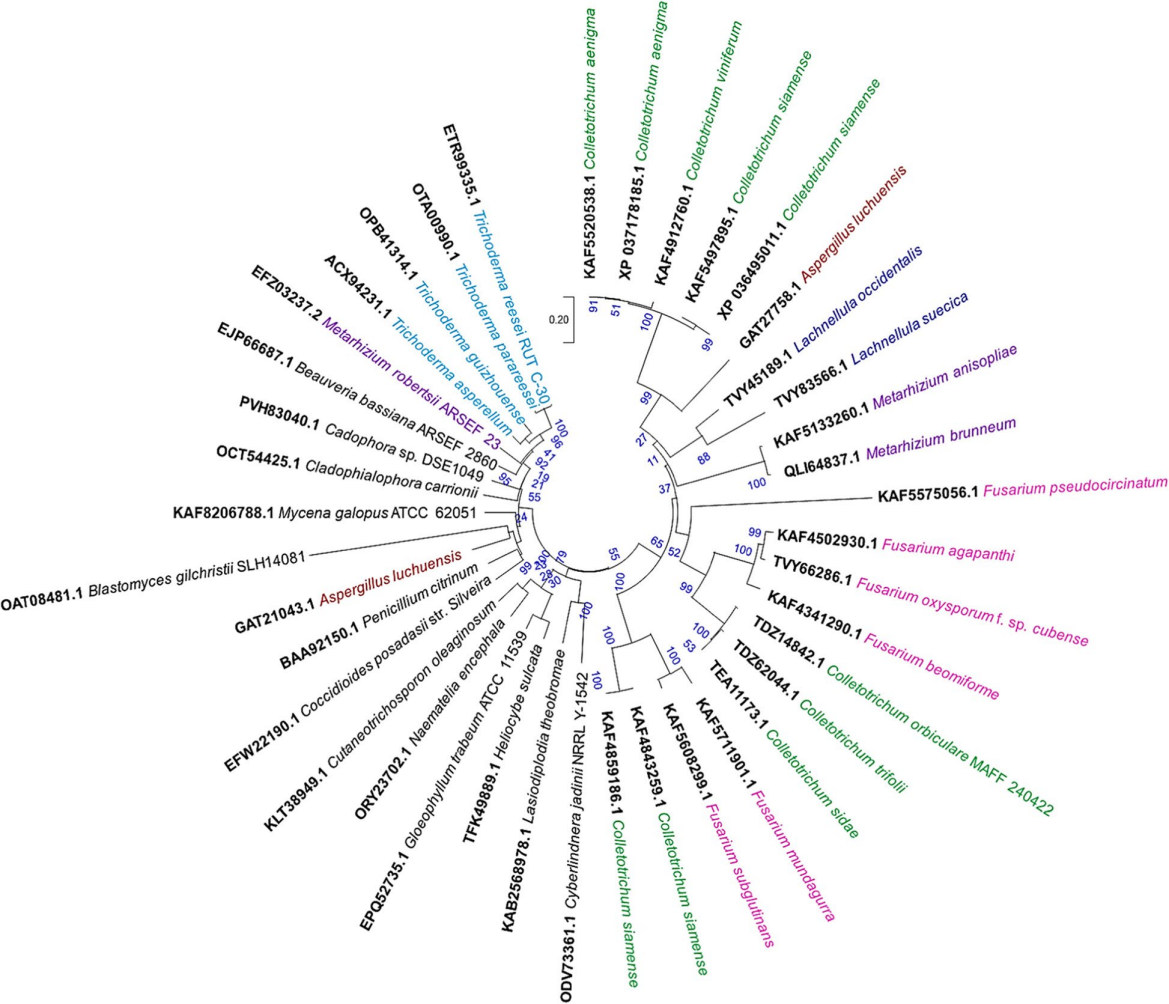
Sequence homology-based phylogenetic tree of selected fungal ACC deaminase sequences. The tree was inferred in MEGA X using the Neighbor-Joining method with 1000 bootstraps. The evolutionary distances were computed using the Poisson correction method

To decode the evolutionary consequences among the selected genera, a phylogenetic tree was constructed based on the fACCD sequences (Fig. 1). The amino acid sequence homology-based phylogeny depicts the clustering pattern among different fungal genera among which *Colletotrichum* spp. occupied the major clades of homologs (Fig. 1). The said genus was found with the closest clustering tendency with the second most abundant genus *Fusarium* spp. in two different clades. The third abundant genus, *Trichoderma*, was however created a separate clade far distant from *Colletotrichum* spp. and *Fusarium* spp. (Fig. 1). Besides, *Metarhizium* spp. and *Lachnellula* spp. showed closer affinity to *Colletotrichum* spp. and *Fusarium* spp. found in between them with two different clades (Fig. 1). Other fungal species, having less frequent in number, was however distributed in the phylogenetic tree with no definitive and inferable pattern (Fig. 1).

### Physicochemical characterization

**Fig. 2:**
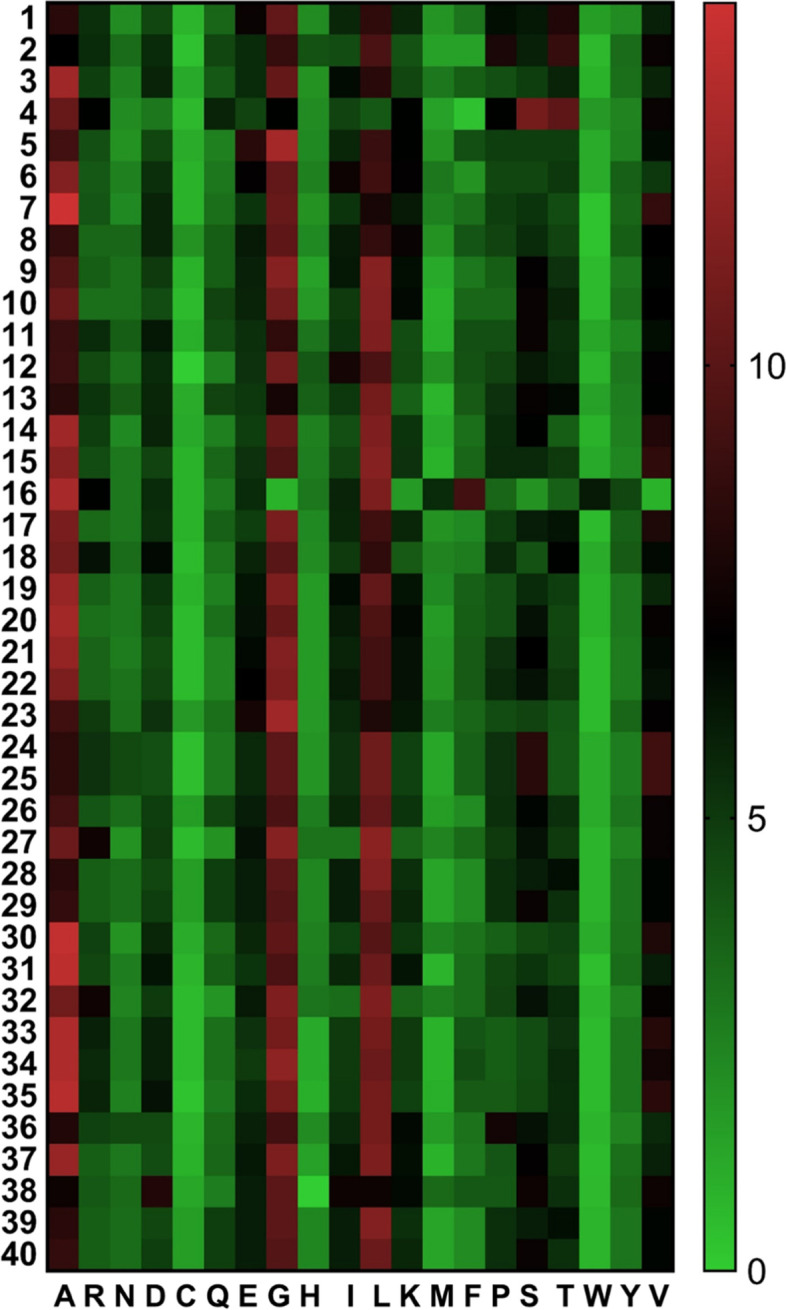
Heatmap analysis depicting variation in amino acid composition among 40 fungal ACC deaminases (ACCNK1 to ACCNK40 are denoted as 1–40 respectively). Amino acids are given in one-letter code. The scale in the right side of heatmap represents percentage of amino acids

Selected 40 fACCD were characterized to depict the theoretical information on physical and chemical features (Table 1, Fig. 2). Heatmap analysis reveals a considerable variation in amino acid composition is noticeable among the fACCD (Fig. 2). The analysis indicated that the linear chain of fACCD proteins has a sequence length ranging from 149 to 750 amino acid residues having molecular weights (MW) between 16.18 and 82.47 kDa (Table 1). The isoelectric points (pI) suggested that enzyme activities would be optimum in a wide range of pH having pI values, i.e., 4.76–10.06 (Table 1). Furthermore, assuming all pairs of Cys residues form cystines, extinction coefficients (EC) were measured (in 280 nm) which were found to be 13,075–90,800 M^− 1^ cm^− 1^ (Table 1). Moreover, instability indices for most of the fACCD were found below 40 while aliphatic indices (AI) were found higher indicating the thermostable nature of the proteins (Table 1). GRAVY values were found lower in every case whereas computed TNR and TPR are presented in Table 1.

**Table 1:** Physicochemical characterization of ACC deaminase proteins of selected fungal strains

Fungal strain	NCBI accession no.	Sequence length	Molecular weight (KDa)	Isoelectric point (pI)	Extinction coefficient (EC) (M^− 1^ cm^− 1^)	Instability index (II)	Aliphatic index (AI)	Grand average of hydropathicity (GRAVY)	Total number of negatively charged residue (TNR)	Total number of positively charged residue (TPR)
*Aspergillus luchuensis*	GAT21043.1	370	40.14	6.28	45,045	39.35	81.49	− 0.319	44	41
*Aspergillus luchuensis*	GAT27758.1	454	49.48	6.34	38,975	43.05	82.25	−0.454	50	44
*Beauveria bassiana* ARSEF 2860	EJP66687.1	346	37.19	5.70	33,140	29.91	87.80	−0.060	39	33
*Blastomyces gilchristii* SLH14081	OAT08481.1	275	29.66	10.06	38,055	53.58	65.67	−0.628	21	38
*Cadophora* sp. DSE1049	PVH83040.1	348	37.51	5.84	35,660	28.96	85.52	−0.168	45	39
*Coccidioides posadasii* str. *Silveira*	EFW22190.1	350	37.90	6.03	41,495	31.66	90.97	−0.181	44	39
*Cutaneotrichosporon oleaginosum*	KLT38949.1	344	36.32	6.19	23,505	30.08	90.26	0.005	38	35
*Cyberlindnera jadinii* NRRL Y-1542	ODV73361.1	344	37.36	6.09	25,245	32.52	86.77	−0.170	41	37
*Fusarium agapanthi*	KAF4502930.1	340	36.14	6.25	26,025	35.39	98.15	−0.044	37	35
*Fusarium beomiforme*	KAF4341290.1	342	36.40	6.61	27,515	28.42	94.74	−0.073	35	34
*Fusarium mundagurra*	KAF5711901.1	631	69.16	6.04	66,725	38.28	92.00	−0.196	73	63
*Fusarium pseudocircinatum*	KAF5575056.1	379	40.98	6.10	32,890	26.40	96.52	−0.012	41	34
*Fusarium subglutinans*	KAF5608299.1	750	82.47	6.06	90,800	39.20	90.77	−0.211	81	67
*Gloeophyllum trabeum* ATCC 11539	EPQ52735.1	346	36.60	6.51	30,160	28.62	96.76	0.021	37	35
*Heliocybe sulcata*	TFK49889.1	339	36.24	6.76	34,045	28.67	99.59	0.035	34	33
*Metarhizium robertsii* ARSEF 23	EFZ03237.2	347	37.06	6.12	31,525	34.67	91.73	−0.023	39	36
*Naematelia encephala*	ORY23702.1	350	37.00	6.08	30,495	24.63	92.29	−0.043	36	32
*Penicillium citrinum*	BAA92150.1	360	39.17	5.56	42,985	33.16	83.25	−0.276	45	37
*Trichoderma asperellum*	ACX94231.1	348	37.06	5.77	31,525	26.35	94.86	0.019	40	35
*Trichoderma guizhouense*	OPB41314.1	345	36.64	5.77	31,525	25.72	94.52	0.011	39	34
*Trichoderma parareesei*	OTA00990.1	359	37.87	5.94	26,025	31.26	90.03	−0.033	40	36
*Trichoderma reesei* RUT C-30	ETR99335.1	358	38.00	5.64	26,025	31.73	89.72	−0.077	42	36
*Lasiodiplodia theobromae*	KAB2568978.1	339	36.63	5.65	29,255	30.44	82.60	−0.233	44	38
*Colletotrichum siamense*	KAF4843259.1	375	40.37	7.76	37,025	38.96	97.23	−0.035	37	38
*Colletotrichum siamense*	KAF4859186.1	375	40.37	7.76	37,025	38.96	97.23	−0.035	37	38
*Colletotrichum viniferum*	KAF4912760.1	368	39.66	5.95	38,765	33.76	93.04	−0.138	40	34
*Metarhizium anisopliae*	KAF5133260.1	358	38.51	6.85	30,035	37.19	89.41	−0.134	41	40
*Colletotrichum siamense*	KAF5497895.1	368	39.60	6.01	33,265	37.32	95.95	−0.117	39	44
*Colletotrichum aenigma*	KAF5520538.1	368	39.55	6.01	33,265	39.21	93.04	−0.166	40	35
*Mycena galopus* ATCC 62051	KAF8206788.1	352	37.69	6.01	38,640	27.39	94.32	0.033	40	35
*Cladophialophora carrionii*	OCT54425.1	367	39.30	6.56	29,005	33.79	94.50	−0.122	42	40
*Metarhizium brunneum*	QLI64837.1	362	38.92	7.20	30,035	36.78	88.70	−0.130	40	40
*Colletotrichum orbiculare* MAFF 240422	TDZ14842.1	340	36.22	6.46	26,025	27.80	98.76	0.039	38	37
*Colletotrichum trifolii*	TDZ62044.1	340	36.05	6.46	26,025	24.54	95.91	0.029	37	36
*Colletotrichum sidae*	TEA11173.1	311	33.02	5.55	24,410	23.82	100.13	0.059	37	33
*Lachnellula occidentalis*	TVY45189.1	357	38.76	8.78	30,035	40.52	88.82	−0.287	37	41
*Fusarium oxysporum* f. sp. *cubense*	TVY66286.1	340	35.92	6.55	27,515	31.95	96.79	−0.030	36	35
*Lachnellula suecica*	TVY83566.1	149	16.18	4.76	13,075	33.92	86.38	−0.133	21	16
*Colletotrichum siamense*	XP_036495011.1	368	39.60	6.01	33,265	37.32	95.95	−0.117	39	34
*Colletotrichum aenigma*	XP_037178185.1	368	39.55	6.01	33,265	39.21	93.04	−0.166	40	35

### Secondary structure prediction

**Fig. 3:**
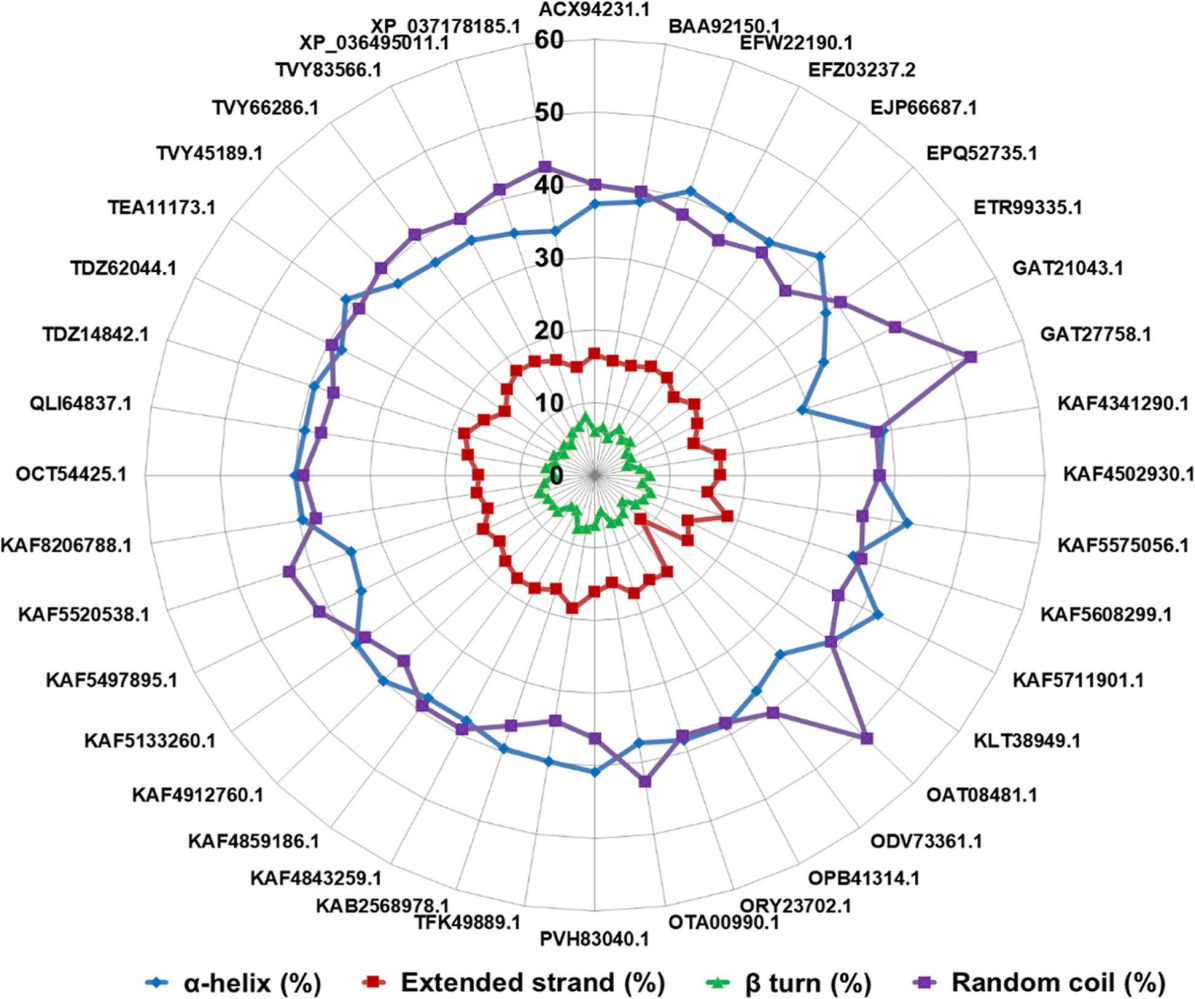
Comparison of predicted secondary structural conformations among 40 fungal ACC deaminases

The secondary structural conformation is the local folded structures that form within a polypeptide chain as a result of interaction among the atoms of the backbone (between the amino hydrogen and carboxyl oxygen atoms). The α-helices and β-sheets are the two most common conformations that indicate the stability of a protein of interest. Here, we analyzed the primary amino acid chains of all fACCD to predict the same. The results suggested that the proteins are abundant in α-helices and random coils while the least contents are shown in the case of extended strands and β-turns (Fig. 3).

### Template selection and homology-based modeling

**Fig. 4:**
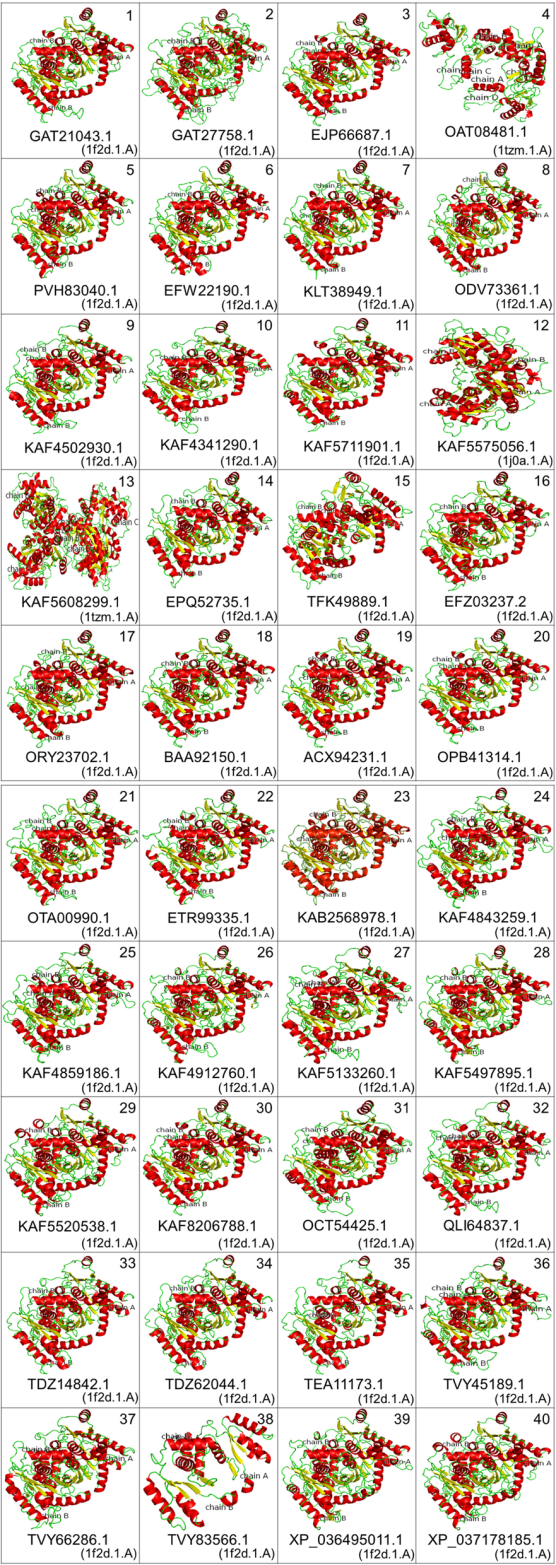
Three-dimensional models of fungal ACC deaminases (red = helix, yellow = sheet, green = loop). Templates for each model are in parenthesis

Structural information is crucial to determine the protein function than merely the primary sequence information. The overall 3-D arrangement of a polypeptide chain is the consequence of the interactions between the polar and charged amino acids. Since homology modeling or comparative protein modeling is a useful tool for the prediction of protein structure, the target-template alignment is important to initiate the task. Collectively, three templates were chosen viz. 1f2d.1.A, 1tzm.1.A, and 1j0a.1.A to perform the homology modeling of 40 fACCD “target” proteins (Fig. 4). The 40 built models with suitable templates were designated consecutively as ACCNK1 to ACCNK40 (Table 2, Fig. 4). It was found that most of the proteins were homo-dimer except ACCNK13 which was a homo-tetramer (Fig. 4).

**Table 2:** Model availability and quality assessment of built fungal ACCD protein models

NCBI accession no.	Model availability		Quality scores			Distribution of amino acid residues in Ramachandran plot (%)	
	Model designation	PMDB ID	QMEAN score	MolProbity score	SAVES ERRAT overall quality factor (%)	Favored region	Outlier region
GAT21043.1	ACCNK1.pdb	PM0083418	−0.74	1.37	91.061	93.62	0.72
GAT27758.1	ACCNK2.pdb	PM0083419	−5.02	2.20	79.739	83.93	5.96
EJP66687.1	ACCNK3.pdb	PM0083420	−0.91	1.71	91.512	92.66	1.05
OAT08481.1	ACCNK4.pdb	PM0083421	−5.49	2.32	91.241	88.99	2.20
PVH83040.1	ACCNK5.pdb	PM0083422	−1.52	1.66	91.730	93.30	1.04
EFW22190.1	ACCNK6.pdb	PM0083423	−1.62	1.78	93.865	91.84	1.78
KLT38949.1	ACCNK7.pdb	PM0083424	−1.18	1.50	91.988	94.61	1.35
ODV73361.1	ACCNK8.pdb	PM0083425	−0.38	1.34	95.173	94.38	0.15
KAF4502930.1	ACCNK9.pdb	PM0083426	−1.83	1.91	90.417	92.42	2.42
KAF4341290.1	ACCNK10.pdb	PM0083427	−2.59	2.05	91.209	89.76	2.45
KAF5711901.1	ACCNK11.pdb	PM0083428	−2.71	1.58	93.924	92.68	1.56
KAF5575056.1	ACCNK12.pdb	PM0083429	−3.68	2.23	84.501	91.13	2.33
KAF5608299.1	ACCNK13.pdb	PM0083430	−2.42	1.42	90.972	93.36	1.34
EPQ52735.1	ACCNK14.pdb	PM0083431	−1.84	1.63	91.706	93.77	0.91
TFK49889.1	ACCNK15.pdb	PM0083432	−1.94	1.75	91.732	93.92	0.76
EFZ03237.2	ACCNK16.pdb	PM0083433	−1.53	1.64	95.846	92.81	1.05
ORY23702.1	ACCNK17.pdb	PM0083434	−1.07	1.73	91.641	92.09	0.60
BAA92150.1	ACCNK18.pdb	PM0083435	−1.67	1.72	93.243	91.06	1.32
ACX94231.1	ACCNK19.pdb	PM0083436	−1.28	1.58	94.479	93.45	0.45
OPB41314.1	ACCNK20.pdb	PM0083437	−1.34	1.45	94.939	93.11	0.75
OTA00990.1	ACCNK21.pdb	PM0083438	−1.25	1.87	87.982	92.46	0.87
ETR99335.1	ACCNK22.pdb	PM0083439	−1.56	1.81	93.016	92.88	1.31
KAB2568978.1	ACCNK23.pdb	PM0083476	−1.05	1.14	93.568	94.16	0.75
KAF4843259.1	ACCNK24.pdb	PM0083477	−3.09	1.91	91.692	88.74	2.63
KAF4859186.1	ACCNK25.pdb	PM0083478	−3.09	1.91	91.692	88.74	2.63
KAF4912760.1	ACCNK26.pdb	PM0083479	−3.08	1.80	86.696	90.64	3.07
KAF5133260.1	ACCNK27.pdb	PM0083480	−2.53	1.79	92.447	91.72	0.87
KAF5497895.1	ACCNK28.pdb	PM0083481	−2.71	1.71	92.163	91.09	2.51
KAF5520538.1	ACCNK29.pdb	PM0083482	−2.53	1.71	91.304	89.83	2.65
KAF8206788.1	ACCNK30.pdb	PM0083483	−1.72	1.69	93.151	93.45	0.89
OCT54425.1	ACCNK31.pdb	PM0083484	−1.79	1.72	94.578	92.59	1.16
QLI64837.1	ACCNK32.pdb	PM0083485	−2.19	1.60	90.505	92.65	1.03
TDZ14842.1	ACCNK33.pdb	PM0083486	−2.03	2.06	83.630	89.12	1.36
TDZ62044.1	ACCNK34.pdb	PM0083487	−1.58	1.62	86.613	91.84	1.21
TEA11173.1	ACCNK35.pdb	PM0083488	−1.43	1.93	87.835	90.23	1.49
TVY45189.1	ACCNK36.pdb	PM0083489	−2.87	2.05	86.982	88.62	2.59
TVY66286.1	ACCNK37.pdb	PM0083490	−2.76	2.34	89.953	90.67	2.45
TVY83566.1	ACCNK38.pdb	PM0083491	−4.84	2.02	88.015	91.84	2.13
XP_036495011.1	ACCNK39.pdb	PM0083492	−2.71	1.71	92.163	91.09	2.51
XP_037178185.1	ACCNK40.pdb	PM0083493	−2.53	1.73	91.304	89.83	2.65

### Structure assessment

The next and essential step of homology-based modeling is the structural validation for the quality of the built protein models. Several quality parameters viz. QMEAN score, MolProbity score, SAVES ERRAT overall quality factor, and distribution of amino acid residues in the Ramachandran plot were taken into consideration to assess the quality of built structures (Table 2). QMEAN and MolProbity score was found numerically lower while an overall quality factor, in most of the cases, were found greater than 90% (Table 2). Also, the distribution of amino acid residues in the Ramachandran plot showed more than 90% of residues occupied in the favored region (Table 2).

### Model deposition

All fACCD, i.e., ACCNK1 to ACCNK40 were finally deposited in the protein model database (PMDB) which stores annotated protein models for further studies. The accession numbers PM0083418–39 and PM0083476–93 were assigned automatically by the server for ACCNK1–ACCNK22 and ACCNK23–ACCNK40 respectively. The models can be accessed anytime from the server using the PMDB identifiers.

### Functional analyses

**Fig. 5:**
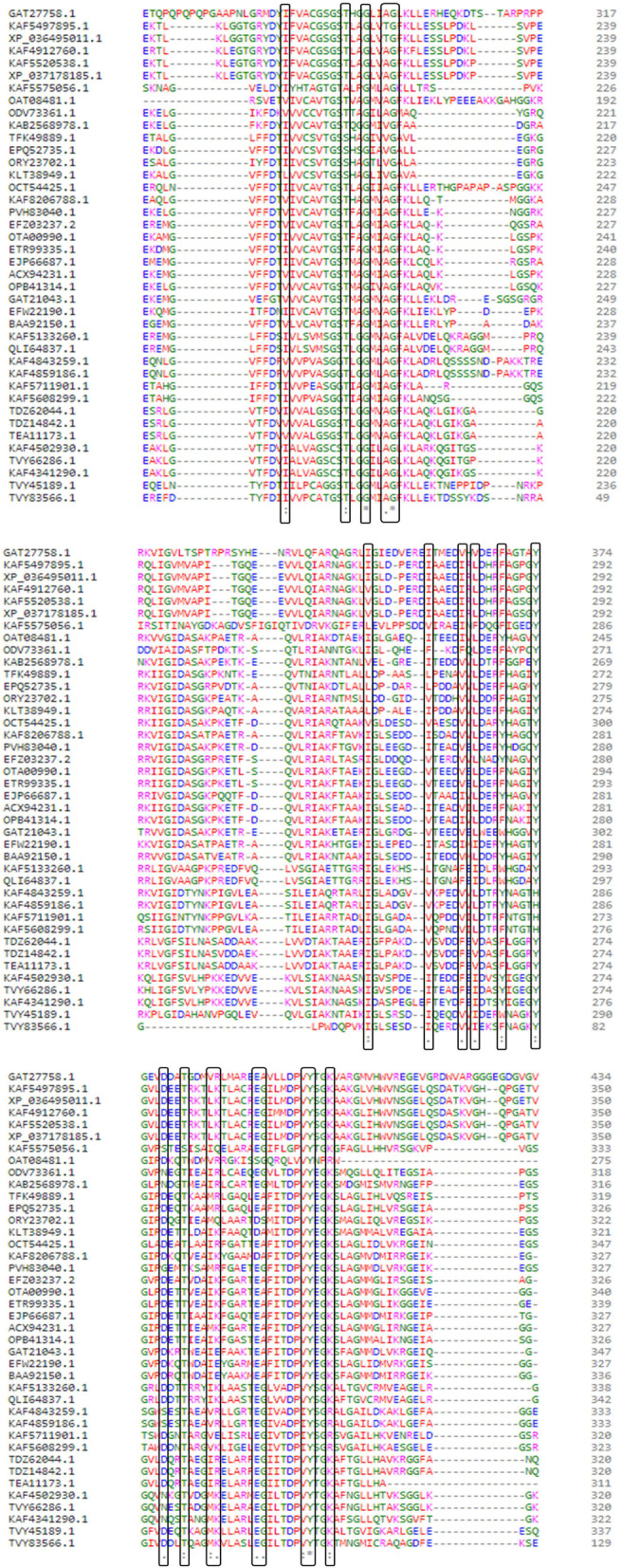
Multiple sequence alignment among 40 fungal ACC deaminases showing conserved amino acid residues. An asterisk (*) in the MSA specified fully conserved residue while a colon (:) indicated conservation between groups of strongly similar properties and a period (.) is the sign of conservation between groups of weakly similar properties

The multiple sequence alignment (MSA) performed through Clustal Omega among all the fACCD recognized several conserved residues within the linear chain of amino acids either fully or partially (Fig. 5). An asterisk (*) in the MSA specified fully conserved residue while a colon (:) indicated conservation between groups of strongly similar properties and a period (.) is the sign of conservation between groups of weakly similar properties (Fig. 5). Besides, from the functional analysis, it was revealed that the proteins contained 1–4 functional motifs (Fig. S3).

## Discussion

Microbial ACC deaminase is an inducible enzyme that can be induced by the presence of its substrate, ACC. ACC has been reported to utilize as a sole source of nitrogen by *Fusarium graminearum* [24] and by a biocontrolling PGPF, *Trichoderma asperellum* T203 [9]. The gene acdS encodes the enzyme *Acd*S which is regulated differentially under different environmental stresses [3]. Unlike bacterial ACC deaminase, the distribution of this enzyme in fungal species is not so frequent. As a result, an in-depth understanding of fungal-derived *Acd*S is lacking. However, few recent investigations successfully amplified the *Acd*S protein sequences from different fungal genera such as *Colletotrichum*, *Fusarium*, *Trichoderma*, *Metarhizium*, *Lachnellula*, *Aspergillus*, *Beauveria*, *Blastomyces*, *Cadophora*, *Cladophialophora*, *Coccidioides*, *Cutaneotrichosporon*, *Cyberlindnera*, *Gleophyllum*, *Heliocybe*, *Mycena*, *Naematelia*, *Lasiodiplodia*, and *Penicillium* (Table 1, Fig. 1, Fig. S1). Interestingly, all the fungal taxa are restricted within the division Ascomycota (74%) and Basidiomycota (26%) (Fig. S2). Nevertheless, further examination is needed to explore the structural and functional characteristics of the said proteins. For this, crystal structure analysis is required which can be performed through biophysical tools either through nuclear magnetic resonance (NMR), X-ray crystallography, or by X-ray free-electron lasers (FELs) [25–27]. The experimental processes are however time-consuming, luxurious, and often difficult to perform in case of a large number of isolated proteins. For the selection and screening from large protein datasets, several bioinformatics tools could be useful to predict protein-folding patterns and 3-D structures as well as generating hypotheses about a protein’s function directing future works on a protein of interest [28].

In this study, we have selected 40 fungal ACC deaminase proteins obtained from 19 different fungal genera from the NCBI database eliminating the ambiguous sequences. Phylogenetic analysis suggested that *Colletotrichum* spp. occupied the major clades of homologs among different fungal genera (Fig. 2). After *Colletotrichum* spp., *Fusarium* spp. and *Trichoderma* spp. are the most dominant genera possessing *Acd*S (Fig. 2). An earlier phylogenetic study on different microbial taxa supported that the acdS genes are predominantly vertically inherited in various fungal classes [8]. Added further, the fACCD sequences were further characterized to decipher the physical and chemical properties that revealed that fACCD are 16.18–82.47 kDa proteins having isoelectric points between 4.76 and 10.06 (Table 1). The isoelectric points below and above the neutral pH are the indication of the acidic and basic nature of the proteins which could be due to the amphoteric nature of amino acid residues [29]. A *Tas-acd*S (ACCD derived from *Trichoderma asperellum*) having 348 amino acids with an expected molecular weight of 37 kDa [9]. Besides, most of fACCD in this study portrayed instability indices < 40 with higher aliphatic indices (relative volume of a protein occupied by aliphatic side chains) which supports their thermostability nature [30, 31]. GRAVY, however, was lower in every case suggesting better interaction with water molecules [31].

Furthermore, selected fACCD were used for secondary structural analysis to uncover the folding pattern of the proteins. This step is crucial as an intermediate state between amino acid sequences and tertiary structures [32]. The results suggested the dominance of α-helical conformation (Fig. 3) indicating protein stability. The α-helices are reported abundant in thermophiles [33]; however, an alike trend was found in the case of phytase proteins of *Aspergillus niger* determined computationally [34]. Likewise, the homology-based protein modeling revealed that fACCD are multimeric proteins, and most of them are homodimeric except ACCNK13, a homotetramer (Fig. 4). The built models for 40 fACCD were sequentially designated as ACCNK1 to ACCNK40 (Table 2). It is accepted that among the approachable prediction methods, homology modeling is the most successful one, for the protein tertiary structure prediction if at least one suitable template (experimentally derived) of the protein family is available in the protein data bank (PDB) [35]. However, the secondary structural elements were found in agreement with the 3D models obtained through homology modeling.

To conform with the reliability of the computed models, several structure-assessment tools were adopted that generated numerical quality scores such as QMEAN score, MolProbity score, SAVES ERRAT overall quality factor, and distribution of amino acid residues in the Ramachandran plot validating the accuracy and stereochemical quality of the structures (Table 2). The QMEAN score should be within 0–1 to obtain high-resolution structures [36] whereas a MolProbity score is a single number that signifies the central MolProbity protein quality statistics, lower the MolProbity score higher the resolution [37]. On the other hand, SAVES ERRAT overall quality factor > 95% determines a high-resolution structure [38]. Also, the distribution of amino acid residues more than 90% in the favored region of Ramachandran plot suggested the characteristics of a good model [39].

Nonetheless, the built protein models in PDB format were deposited to the protein model database (PMDB) with accession numbers PM0083418–39 and PM0083476–93 for further use. Finally, the functional annotation from Clustal Omega evidenced the conserved residues for the selected 40 fACCD (Fig. 5). Conserved residues in proteins have an important role in protein folding and unfolding kinetics and protein stability as well [40]. Furthermore, the motif search result indicated that the common motif shared by all proteins were “PALP” which suggested the proteins belong to pyridoxal-phosphate dependent class of enzymes (Fig. S3).

## Conclusions

There was a dire need to assemble fungal ACC deaminase protein sequences (fACCD) derived experimentally in order to decipher the physicochemical, stereochemical, and functional features for a comprehensive overview. Keeping in mind the constraints in utilizing the modern biophysical tools to obtain crystal structures, computational annotation was proven useful to predict the structure-function aspects of proteins of interest. This study unveils the characterization of fACCD indicating that these are multimeric proteins having molecular weight 16–82 kDa, with both acidic and alkaline property and thermostable in nature. To date, the fACCD are predominant in different genera of Basidiomycota followed by Ascomycota. It is important to note that among the Asco- and Basidiomycota members, both beneficial and phytopathogens possess this plant growth promoting enzyme. This might be due to an evolutionary consequence which may serve as a meaningful cue in symbiotic as well as host-pathogen interaction studies. Thus, as an integral part of in vitro studies, anticipated features of fungal ACC deaminase would direct to design, identify, and engineer novel variants of this plant-stress related fungal protein for their effective application in industrial-scale.

## Supplementary Information

**Additional file 1: Table S1.** Amino acid composition of selected fACCD proteins. **Fig. S1.** Abundance of fungal species possessing ACC deaminase. **Fig. S2.** Classification of 40 fungal taxa possessing ACC deaminase into divisions. **Fig. S3.** Functional motifs found in selected fungal ACCD proteins.

## Data Availability

The amino acid sequences used in this study for computational analyses are available at NCBI database. The built protein models are available at PMDB database. The sequence and model accession numbers are given in Tables 1 and 2.

## Abbreviations

ACCD: 1-Aminocyclopropane-1-carboxylic acid (ACC) deaminase
fACCD: Fungal ACC deaminase
PMDB: Protein model database
GRAVY: Grand average of hydropathicity
BLAST: Basic Local Alignment Search Tool
GMQE: Global model quality estimation
QSQE: Quaternary structure quality estimation
SOPMA: Self-optimized prediction method with alignment
