# Supplementary material for: Structural heterogeneity assessment among the isoforms of fungal 1-aminocyclopropane-1-carboxylic acid (ACC) deaminase: a comparative in silico perspective
Source: J Genet Eng Biotechnol. 2022 Feb 1;20:18. doi: 10.1186/s43141-021-00294-0 (PMC8807812; doi:10.1186/s43141-021-00294-0)
Supplement: Supplementary file 1 — Additional file 1: Table S1. Amino acid composition of selected fACCD proteins. Fig. S1. Abundance of fungal species possessing ACC deaminase. Fig. S2. Classification of 40 fungal taxa possessing ACC deaminase into divisions. Fig. S3. Functional motifs found in selected fungal ACCD proteins. [file 43141_2021_294_MOESM1_ESM.docx]

**Table S1.** Amino acid composition of selected fACCD proteins.

| Protein acc. | Ala (A) | Arg (R) | Asn (N) | Asp (D) | Cys (C) | Gln (Q) | Glu (E) | Gly (G) | His (H) | Ile (I) | Leu (L) | Lys (K) | Met (M) | Phe (F) | Pro (P) | Ser (S) | Thr (T) | Trp (W) | Tyr (Y) | Val (V) |
| --- | --- | --- | --- | --- | --- | --- | --- | --- | --- | --- | --- | --- | --- | --- | --- | --- | --- | --- | --- | --- |
| GAT21043.1 | 8.4 | 5.4 | 2.2 | 4.6 | 0.8 | 3.5 | 7.3 | 10.3 | 2.2 | 5.7 | 8.6 | 5.7 | 1.9 | 3.0 | 6.5 | 6.2 | 8.1 | 1.6 | 2.2 | 5.9 |
| GAT27758.1 | 7.0 | 5.5 | 3.3 | 5.5 | 0.4 | 4.6 | 5.5 | 8.8 | 4.2 | 4.4 | 9.5 | 4.2 | 1.5 | 1.5 | 7.9 | 5.9 | 8.8 | 0.7 | 3.3 | 7.3 |
| EJP66687.1 | 12.4 | 4.9 | 2.6 | 5.8 | 1.2 | 4 | 5.5 | 10.4 | 2 | 6.6 | 8.4 | 4.6 | 2.9 | 3.8 | 4.3 | 4.9 | 5.8 | 0.9 | 3.2 | 5.8 |
| OAT08481.1 | 10.5 | 6.9 | 2.2 | 2.9 | 0.7 | 5.8 | 4.7 | 6.9 | 2.2 | 4.7 | 4 | 6.9 | 1.5 | 0.4 | 6.9 | 10.9 | 10.2 | 1.8 | 2.5 | 7.3 |
| PVH83040.1 | 9.2 | 4.3 | 2 | 4.6 | 1.1 | 2.6 | 8.3 | 12.6 | 2.3 | 5.7 | 8.9 | 6.9 | 2 | 4.3 | 4.9 | 4.9 | 4.9 | 1.1 | 2.6 | 6.6 |
| EFW22190.1 | 11.4 | 4 | 2.6 | 5.4 | 0.9 | 2.9 | 7.1 | 10.3 | 2.6 | 7.4 | 9.1 | 7.1 | 2.9 | 2 | 4.6 | 4.6 | 5.1 | 1.1 | 3.7 | 5.1 |
| KLT38949.1 | 14 | 4.1 | 2.3 | 5.8 | 0.9 | 3.2 | 5.2 | 10.5 | 2 | 5.2 | 7.8 | 6.1 | 2.6 | 3.2 | 4.9 | 5.2 | 4.4 | 0.3 | 3.5 | 8.7 |
| ODV73361.1 | 8.7 | 3.5 | 3.5 | 5.8 | 2 | 3.8 | 6.1 | 10.2 | 2.3 | 6.1 | 8.7 | 7.3 | 2 | 4.1 | 4.7 | 5.5 | 4.7 | 0.3 | 3.8 | 7 |
| KAF4502930.1 | 9.7 | 3.8 | 3.2 | 5 | 0.9 | 3.8 | 5.9 | 11.5 | 1.5 | 6.2 | 11.5 | 6.5 | 1.2 | 2.9 | 3.8 | 7.1 | 5.3 | 0.6 | 2.9 | 6.8 |
| KAF4341290.1 | 10.5 | 3.2 | 3.2 | 4.4 | 0.6 | 4.7 | 5.8 | 10.8 | 1.8 | 5 | 11.4 | 6.7 | 0.9 | 3.5 | 3.5 | 7.3 | 5.8 | 0.6 | 3.2 | 7 |
| KAF5711901.1 | 8.9 | 5.5 | 3.8 | 6.2 | 1 | 4.4 | 5.4 | 8.6 | 3 | 5.2 | 11.3 | 4.4 | 1 | 4.3 | 4.3 | 7.3 | 5.4 | 1.3 | 2.4 | 6.5 |
| KAF5575056.1 | 9 | 4.5 | 3.2 | 5.5 | 0 | 2.6 | 5.3 | 10.8 | 4 | 7.7 | 9.5 | 4.5 | 2.1 | 4.2 | 4.7 | 6.1 | 5.5 | 0.8 | 2.9 | 7.1 |
| KAF5608299.1 | 8.3 | 5.2 | 3.9 | 5.7 | 1.1 | 4.7 | 5.1 | 7.7 | 3.7 | 5.1 | 10.9 | 3.7 | 0.8 | 3.9 | 5.3 | 7.2 | 6.7 | 1.5 | 2.7 | 6.9 |
| EPQ52735.1 | 12.4 | 4.9 | 2.3 | 5.8 | 1.2 | 2.6 | 4.9 | 10.4 | 2.6 | 4.3 | 11.3 | 5.2 | 1.2 | 3.2 | 5.5 | 6.9 | 3.8 | 0.9 | 2.6 | 8.1 |
| TFK49889.1 | 11.5 | 4.4 | 2.9 | 4.7 | 0.9 | 3.5 | 5.3 | 9.7 | 2.7 | 4.7 | 11.5 | 5.3 | 0.9 | 3.5 | 5.6 | 5.6 | 5 | 1.2 | 2.4 | 8.6 |
| EFZ03237.2 | 12.7 | 6.9 | 2.9 | 5.5 | 0.9 | 2.9 | 5.5 | 0.9 | 2.9 | 5.8 | 11.2 | 1.7 | 5.5 | 9.2 | 3.5 | 2 | 3.7 | 6.1 | 4.6 | 0.9 |
| ORY23702.1 | 11.1 | 3.4 | 2.9 | 5.4 | 0.9 | 3.7 | 4.9 | 11.1 | 2.3 | 5.7 | 9.1 | 5.7 | 2 | 2.3 | 4.9 | 6 | 6.3 | 0.6 | 3.7 | 8 |
| BAA92150.1 | 10.8 | 6.4 | 3.3 | 6.7 | 0.6 | 3.1 | 5.8 | 10 | 2.2 | 5 | 8.6 | 3.9 | 2.5 | 2.8 | 5.6 | 4.2 | 6.9 | 1.1 | 3.9 | 6.7 |
| ACX94231.1 | 12.1 | 3.7 | 2.9 | 5.2 | 0.9 | 2.6 | 6.3 | 11.2 | 1.7 | 6.6 | 10.3 | 6.3 | 2.3 | 3.7 | 4.3 | 5.5 | 4.9 | 0.9 | 2.9 | 5.7 |
| OPB41314.1 | 12.5 | 3.2 | 2.9 | 4.9 | 0.6 | 3.2 | 6.4 | 10.4 | 1.7 | 6.1 | 9.6 | 6.7 | 1.7 | 3.8 | 4.3 | 6.4 | 4.6 | 0.9 | 2.9 | 7.2 |
| OTA00990.1 | 12 | 3.6 | 2.8 | 4.5 | 0.6 | 2.5 | 6.7 | 11.4 | 1.7 | 5.8 | 9.2 | 6.4 | 1.9 | 3.9 | 5.3 | 7 | 4.7 | 0.6 | 2.8 | 6.7 |
| ETR99335.1 | 11.2 | 3.6 | 3.1 | 4.7 | 0.6 | 2.5 | 7 | 11.2 | 1.7 | 6.1 | 9.2 | 6.4 | 2 | 3.9 | 5.6 | 6.4 | 5 | 0.6 | 2.8 | 6.4 |
| KAB2568978.1 | 9.1 | 5 | 3.2 | 5.3 | 1.8 | 3.2 | 7.7 | 12.4 | 1.8 | 5.6 | 8 | 6.2 | 2.7 | 3.5 | 4.4 | 4.7 | 4.1 | 0.6 | 3.5 | 7.1 |
| KAF4843259.1 | 8.5 | 5.3 | 4.5 | 4.3 | 0.5 | 2.9 | 5.6 | 10.1 | 1.9 | 5.3 | 10.7 | 4.8 | 1.3 | 3.7 | 5.3 | 8.3 | 4 | 1.1 | 2.7 | 9.1 |
| KAF4859186.1 | 8.5 | 5.3 | 4.5 | 4.3 | 0.5 | 2.9 | 5.6 | 10.1 | 1.9 | 5.3 | 10.7 | 4.8 | 1.3 | 3.7 | 5.3 | 8.3 | 4 | 1.1 | 2.7 | 9.1 |
| KAF4912760.1 | 9.2 | 4.1 | 3.3 | 4.9 | 1.6 | 4.6 | 6 | 9.5 | 2.7 | 5.7 | 10.3 | 5.2 | 1.6 | 2.2 | 5.4 | 6.8 | 5.4 | 1.1 | 3 | 7.3 |
| KAF5133260.1 | 10.6 | 7.5 | 2 | 5 | 0.6 | 2 | 6.4 | 11.5 | 3.1 | 3.1 | 11.7 | 3.6 | 2.5 | 3.4 | 5 | 6.4 | 5 | 0.8 | 2.5 | 7.3 |
| KAF5497895.1 | 8.4 | 3.8 | 3.3 | 4.6 | 1.6 | 4.9 | 6 | 10.1 | 2.4 | 6 | 11.4 | 5.4 | 1.4 | 2.2 | 5.4 | 6 | 6.5 | 0.8 | 3 | 6.8 |
| KAF5520538.1 | 8.7 | 3.8 | 3.3 | 4.9 | 1.6 | 4.9 | 6 | 9.8 | 2.4 | 6 | 10.6 | 5.7 | 1.4 | 2.2 | 5.4 | 7.3 | 5.4 | 0.8 | 3 | 6.8 |
| KAF8206788.1 | 13.6 | 4.8 | 2 | 5.7 | 1.1 | 3.4 | 5.7 | 10.2 | 2.6 | 4.8 | 9.9 | 5.1 | 2.6 | 3.1 | 3.7 | 4.5 | 4.8 | 1.1 | 3.1 | 8 |
| OCT54425.1 | 13.4 | 4.6 | 2.7 | 6.3 | 0.8 | 3.8 | 5.2 | 9.5 | 2.7 | 5.7 | 10.6 | 6.3 | 0.8 | 3.3 | 4.6 | 5.2 | 4.6 | 0.5 | 3.3 | 6 |
| QLI64837.1 | 10.8 | 7.5 | 2.5 | 5 | 0.6 | 1.9 | 6.1 | 11.3 | 3 | 3.3 | 11.3 | 3.6 | 2.8 | 3.3 | 4.7 | 6.4 | 5.5 | 0.8 | 2.5 | 7.2 |
| TDZ14842.1 | 12.9 | 5.9 | 2.9 | 5.9 | 0.6 | 3.2 | 5.3 | 10.9 | 1.2 | 5 | 10.9 | 5 | 0.9 | 4.1 | 3.8 | 4.4 | 5.3 | 0.6 | 2.9 | 8.2 |
| TDZ62044.1 | 12.9 | 5.6 | 2.9 | 5.9 | 0.6 | 3.2 | 5 | 11.8 | 1.2 | 5 | 10.6 | 5 | 0.9 | 4.4 | 3.8 | 4.4 | 5.6 | 0.6 | 2.9 | 7.6 |
| TEA11173.1 | 13.2 | 5.8 | 2.6 | 6.4 | 0.3 | 2.9 | 5.5 | 10.9 | 1 | 5.1 | 10.9 | 4.8 | 1 | 3.9 | 3.9 | 4.5 | 5.5 | 0.6 | 2.9 | 8.4 |
| TVY45189.1 | 8.1 | 4.8 | 4.5 | 4.5 | 0.8 | 3.4 | 5.9 | 9.2 | 2.2 | 5.6 | 10.9 | 6.7 | 1.7 | 3.1 | 7.6 | 6.4 | 5.6 | 0.8 | 2.5 | 5.6 |
| TVY66286.1 | 12.1 | 3.8 | 2.9 | 4.4 | 0.9 | 3.5 | 6.2 | 11.2 | 1.5 | 6.2 | 11.2 | 6.5 | 0.9 | 2.9 | 4.1 | 7.1 | 5 | 0.6 | 3.2 | 5.9 |
| TVY83566.1 | 7.4 | 4 | 3.4 | 8.1 | 1.3 | 2.7 | 6 | 10.1 | 0 | 7.4 | 7.4 | 6.7 | 3.4 | 4 | 4 | 7.4 | 5.4 | 0.7 | 3.4 | 7.4 |
| XP_036495011.1 | 8.4 | 3.8 | 3.3 | 4.6 | 1.6 | 4.9 | 6 | 10.1 | 2.4 | 6 | 11.4 | 5.4 | 1.4 | 2.2 | 5.4 | 6 | 6.5 | 0.8 | 3 | 6.8 |
| XP_037178185.1 | 8.7 | 3.8 | 3.3 | 4.9 | 1.6 | 4.9 | 6 | 9.8 | 2.4 | 6 | 10.6 | 5.7 | 1.4 | 2.2 | 5.4 | 7.3 | 5.4 | 0.8 | 3 | 6.8 |


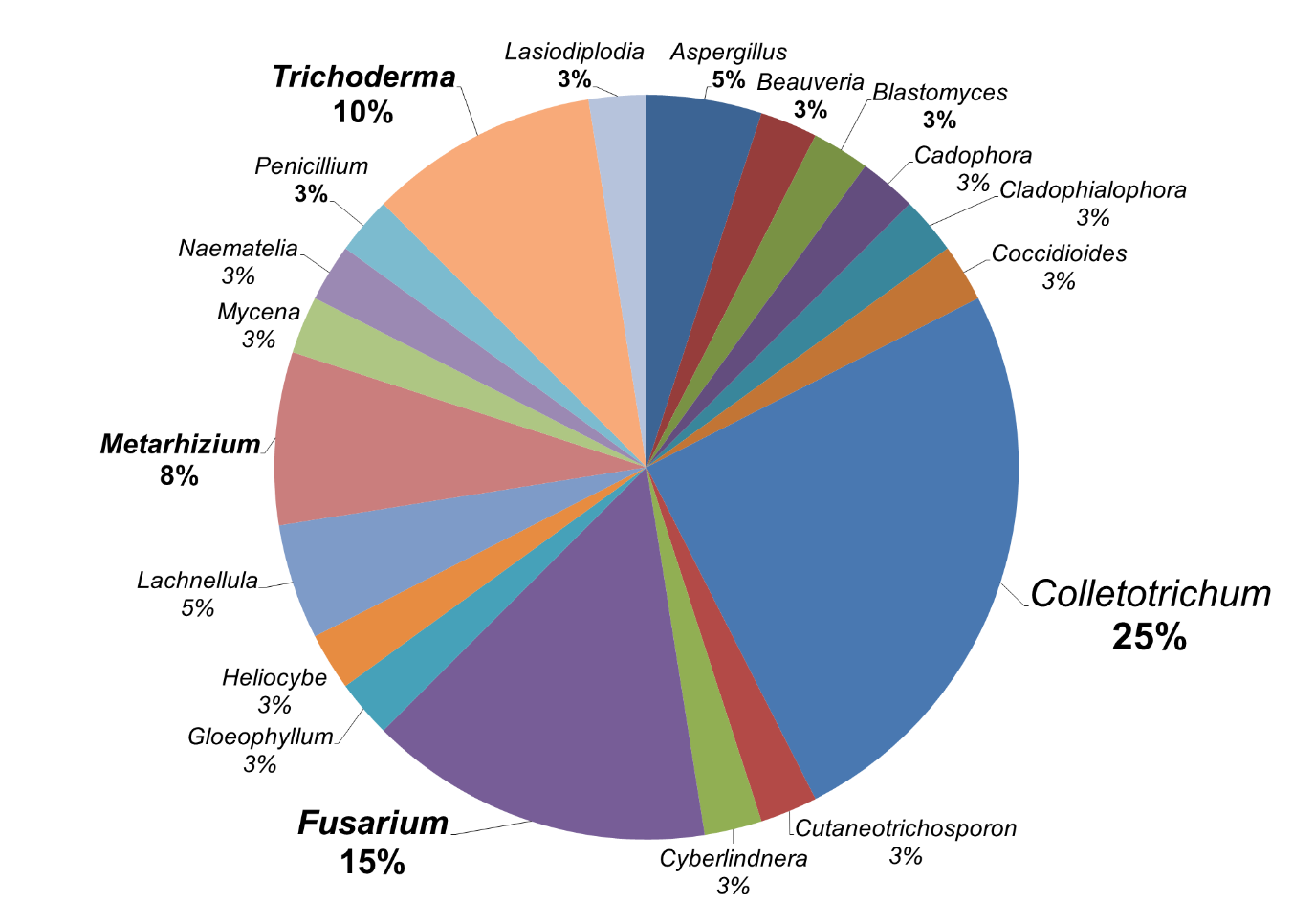


**Fig. S1** Abundance of fungal species possessing ACC deaminase.


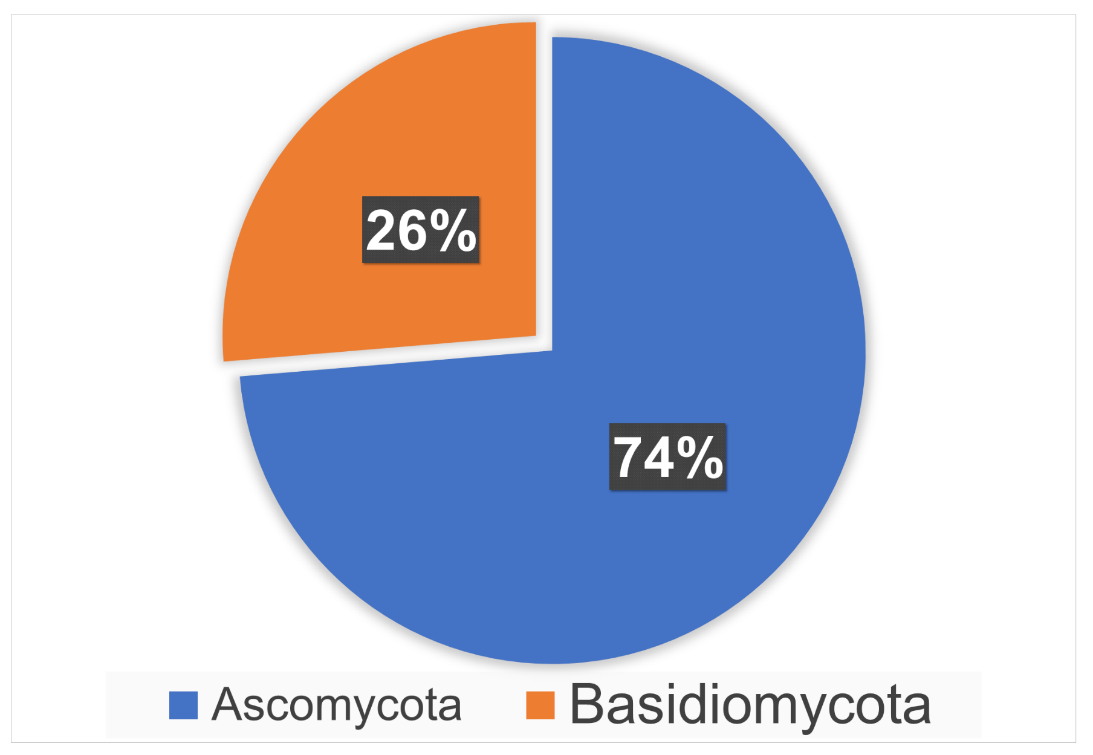


**Fig. S2** Classification of 40 fungal taxa possessing ACC deaminase into divisions.


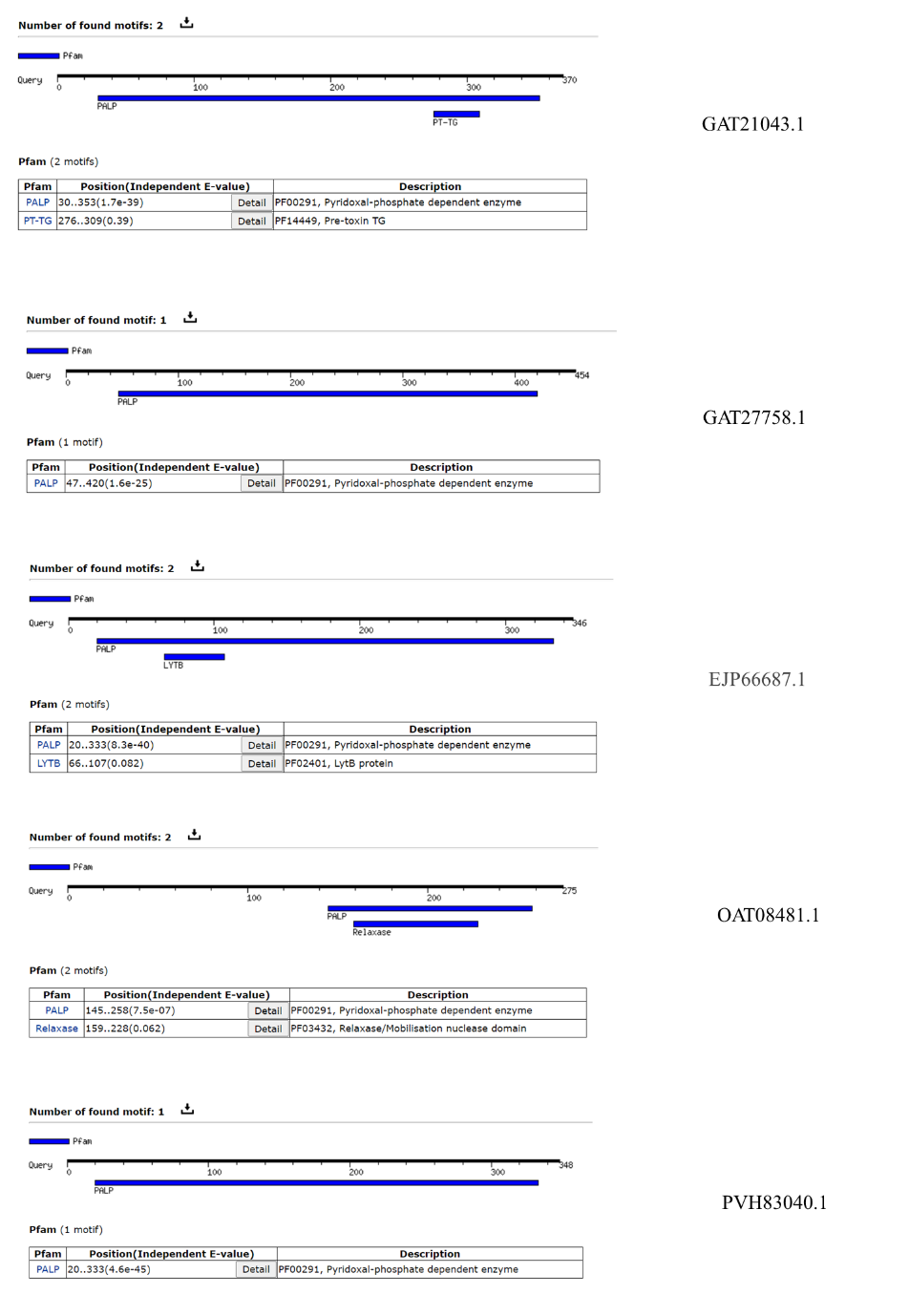

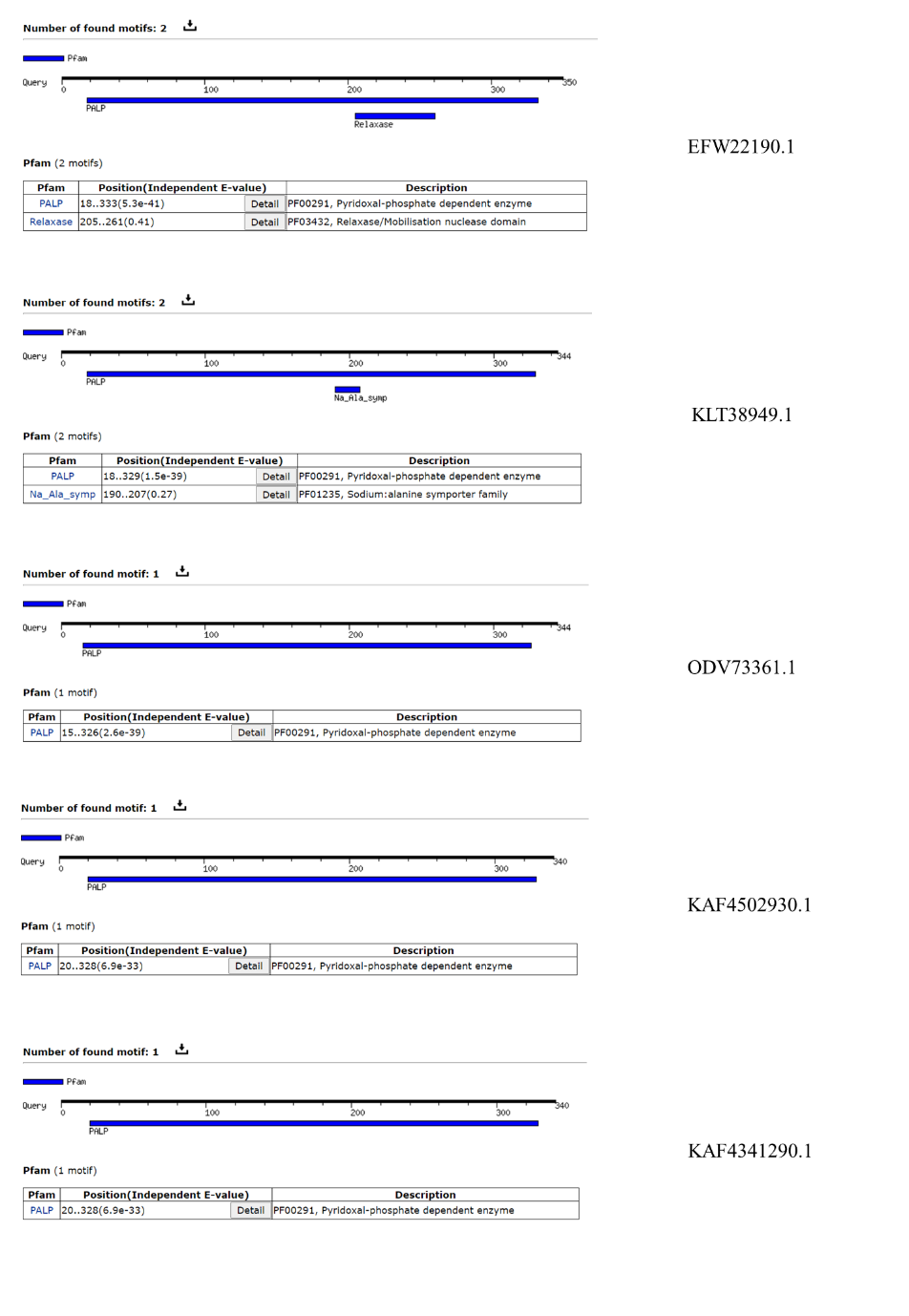


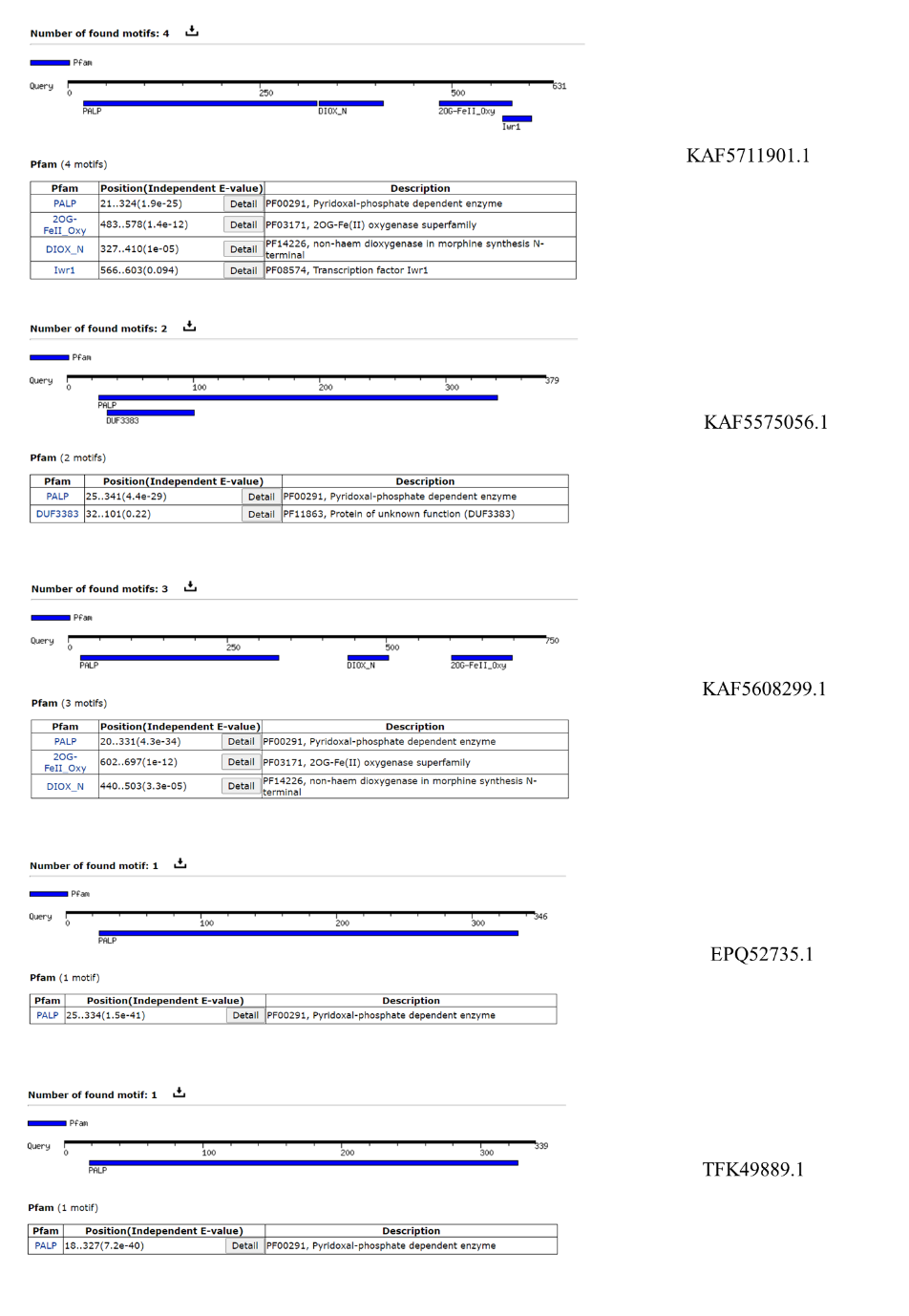

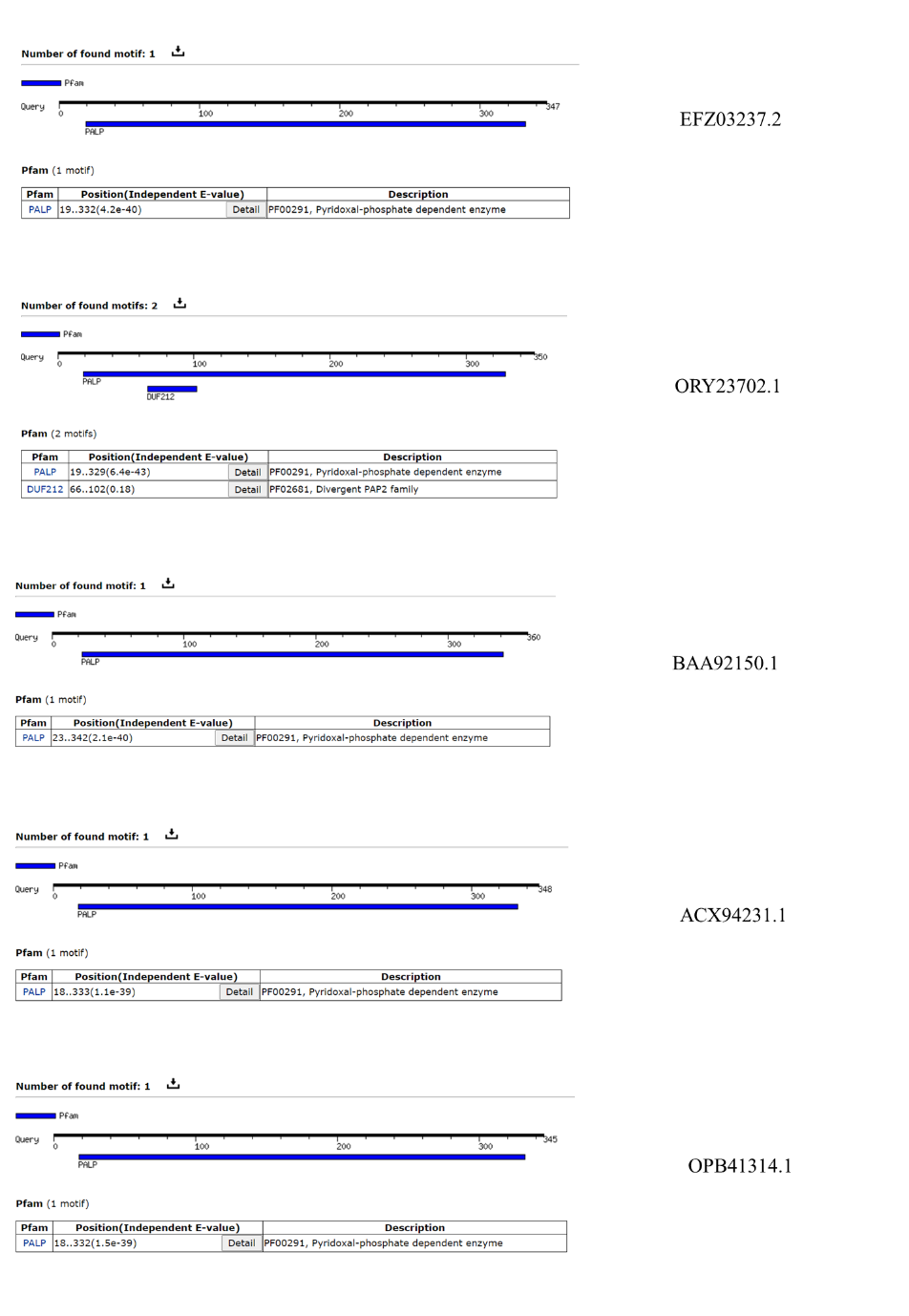


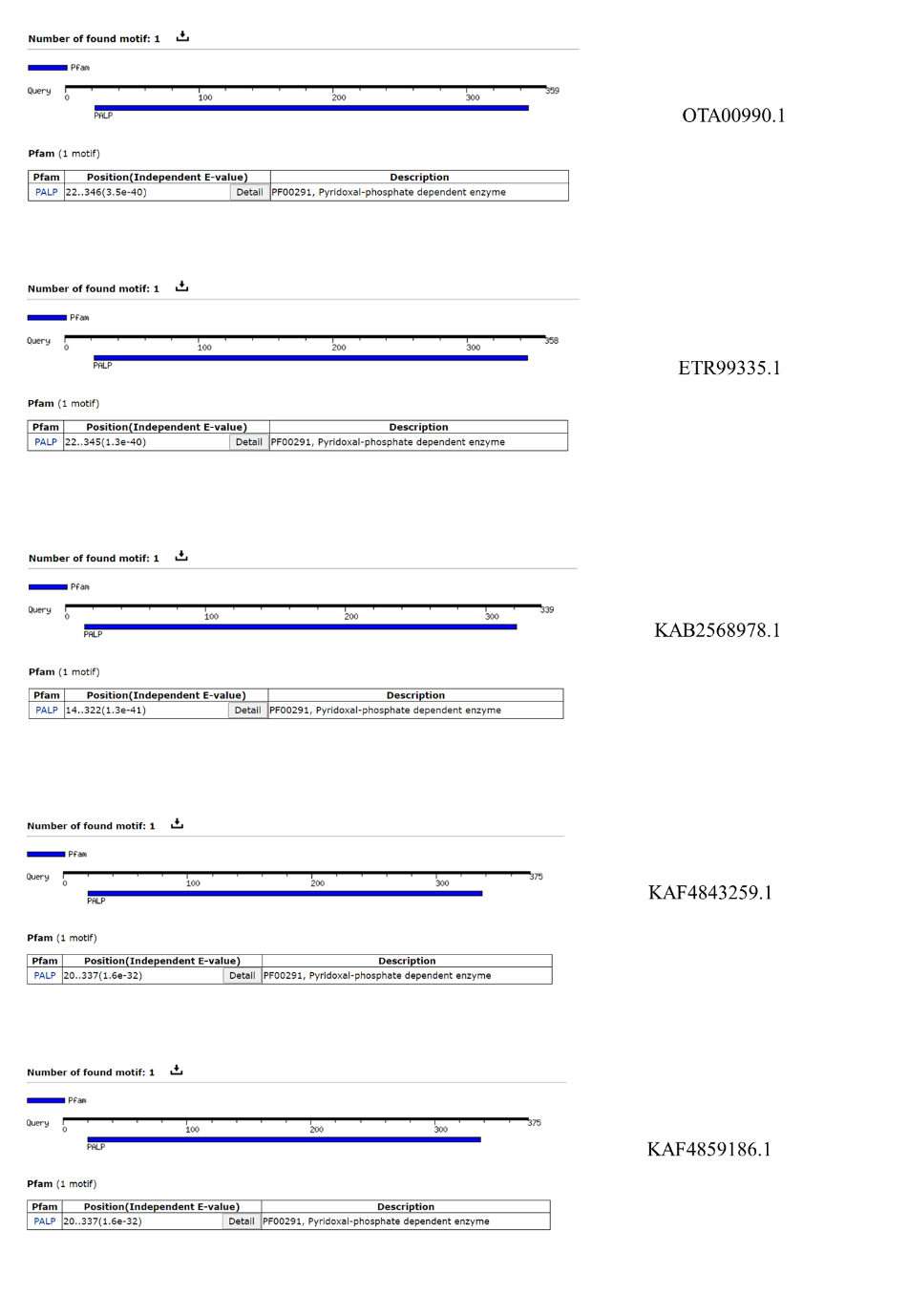

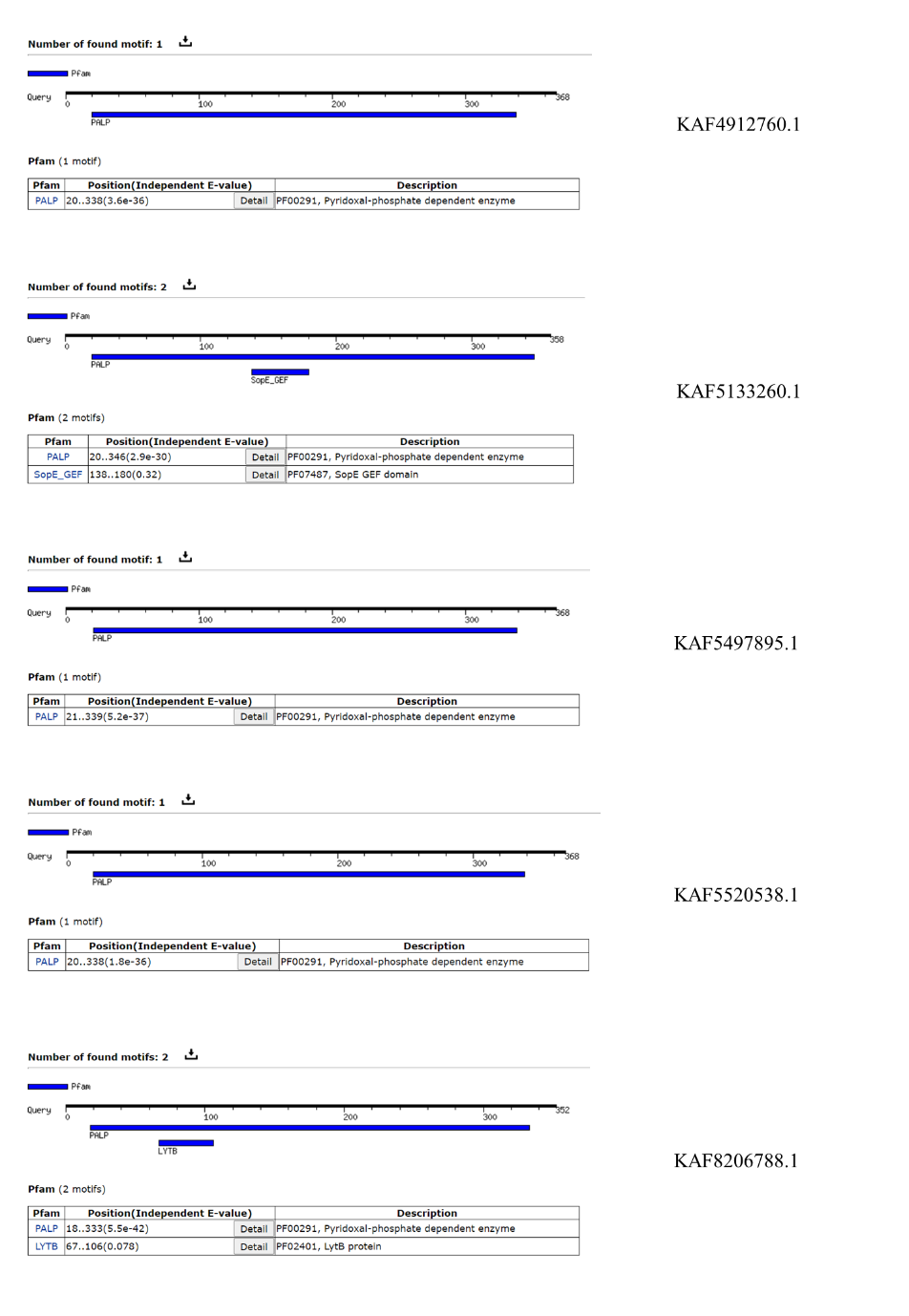


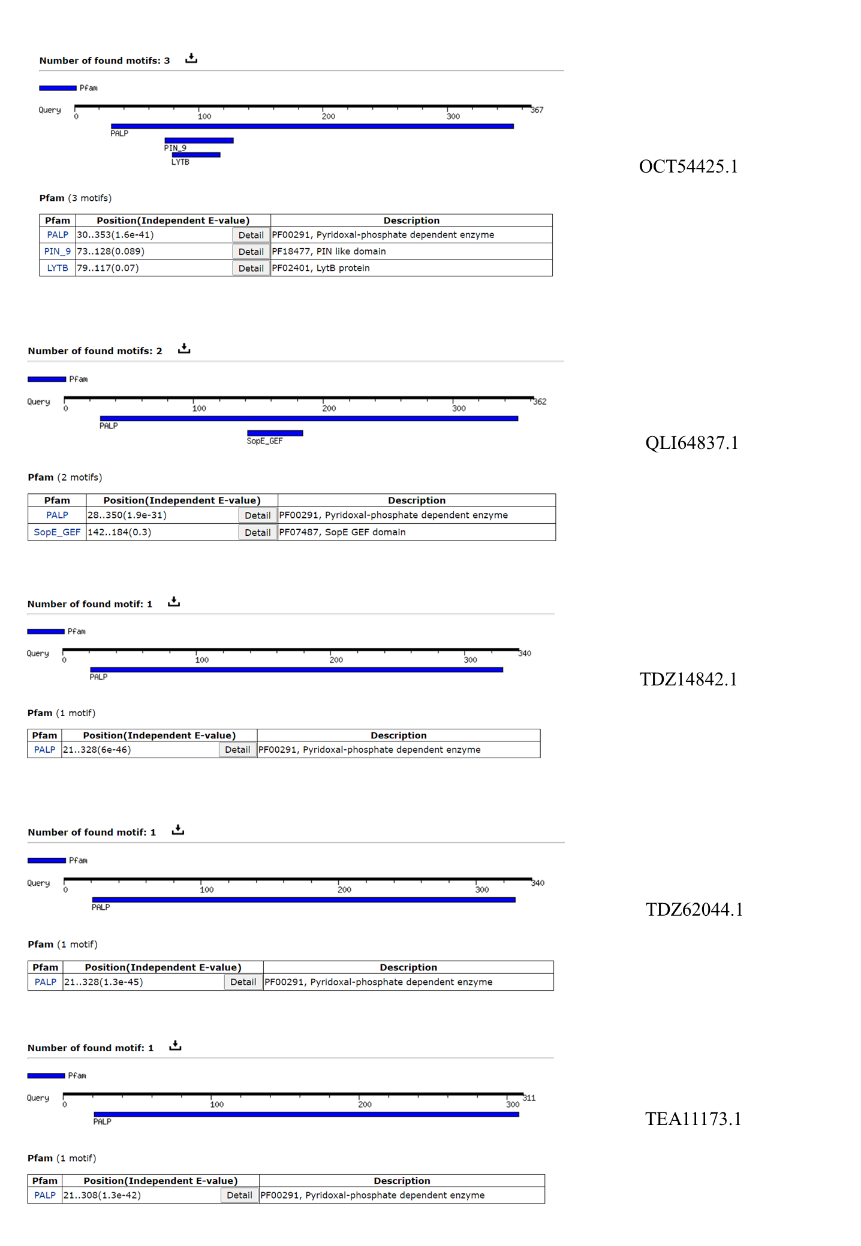

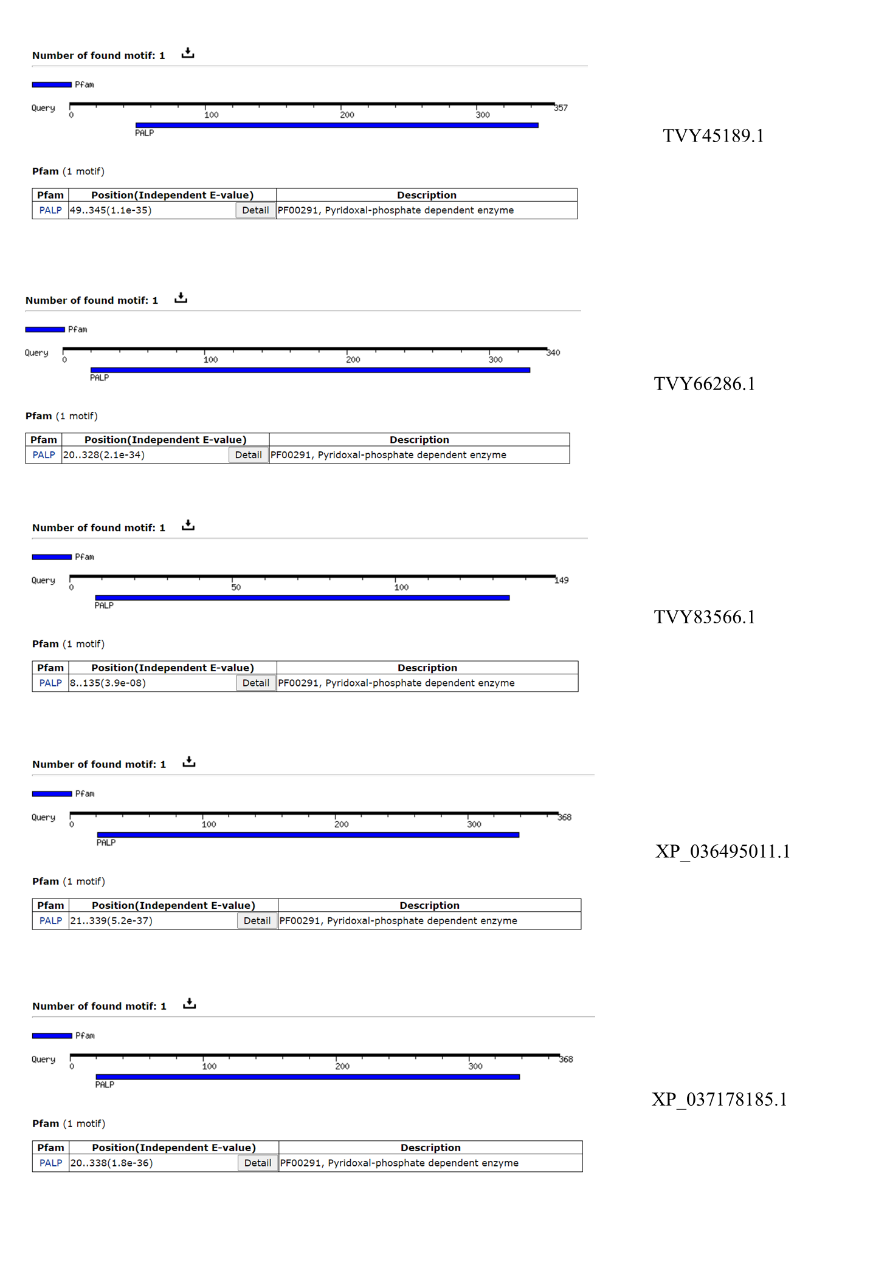


**Fig. S3** Functional motifs found in selected fungal ACCD proteins.
